# Overactive type 2 cannabinoid receptor induces meiosis in fetal gonads and impairs ovarian reserve

**DOI:** 10.1038/cddis.2017.496

**Published:** 2017-10-05

**Authors:** Emanuela De Domenico, Federica Todaro, Gabriele Rossi, Susanna Dolci, Raffaele Geremia, Pellegrino Rossi, Paola Grimaldi

**Affiliations:** 1 Department of Biomedicine and Prevention, University of Rome Tor Vergata, Rome 00133, Italy

## Abstract

Type 2 cannabinoid receptor (CB_2_R) has been proposed to promote *in vitro* meiotic entry of postnatal male germ cells and to maintain the temporal progression of spermatogenesis *in vivo.* However, no information is presently available on the role played by CB_2_R in male and female fetal gonads. Here we show that *in vitro* pharmacological stimulation with JWH133, a CB_2_R agonist, induced activation of the meiotic program in both male and female fetal gonads. Upon stimulation, gonocytes initiated the meiotic program but became arrested at early stages of prophase I, while oocytes showed an increased rate of meiotic entry and progression toward more advanced stage of meiosis. Acceleration of meiosis in oocytes was accompanied by a strong increase in the percentage of *γ*-H2AX-positive pachytene and diplotene cells, paralleled by an increase of TUNEL-positive cells, suggesting that DNA double-strand breaks were not correctly repaired during meiosis, leading to oocyte apoptosis. Interestingly, *in vivo* pharmacological stimulation of CB_2_R in fetal germ cells through JWH133 administration to pregnant females caused a significant reduction of primordial and primary follicles in the ovaries of newborns with a consequent depletion of ovarian reserve and reduced fertility in adult life, while no alterations of spermatogenesis in the testis of the offspring were detected. Altogether our findings highlight a pro-meiotic role of CB_2_R in male and female germ cells and suggest that the use of cannabis in pregnant female might represent a risk for fertility and reproductive lifespan in female offspring.

Meiosis is a crucial event in mammalian reproduction that occurs at different developmental ages in male and female gonads. In the mouse ovary, oogonia enter meiosis during fetal development at E13.5 and they remain arrested at diplotene stage of prophase I until birth. Oocytes complete the first meiotic division only at puberty, upon hormonal stimulation. In the testis, gonocytes remain quiescent for the entire fetal period^1, 2^ resuming proliferation just after birth and initiate meiosis at around 7 days *post natum* (dpn). Retinoic acid (RA) has been proposed as a meiosis-inducing substance, both in fetal female germ cells and in postnatal male germ cells.^3, 4, 5^ RA physiologically induces meiosis in female oocytes during fetal life,^3, 4^ whereas the same action in fetal gonocytes is prevented by the presence of the retinoid-degrading enzyme CYP26B1.^3, 6^ Indeed, in Cyp26b1-null fetal testis, in which RA is elevated, gonocytes enter meiosis.^6^ RA acts by upregulating *Stra8* gene expression in oogonia and in postnatal spermatogonia. *Stra8* is required for meiotic initiation in both sexes; indeed *Stra8*-deficient germ cells in postnatal males and in fetal females arrest just before meiosis, without entering meiotic prophase.^7^ However, conflicting results exist regarding its absolute requirement for the proper germ cell meiotic onset^8^ and other extrinsic and intrinsic factors are likely involved. Recent findings indicate that the endocannabinoid system (ECS) has a role in regulating meiotic entry of isolated postnatal male germ cells. The ECS has been shown to be implicated in several important physiological functions^9, 10^ and it works through two best-characterized cannabinoid receptors, type 1 (CB_1_) and 2 (CB_2_). The two best-known ligands are the endocannabinoids anandamide and 2-arachidonoylglycerol (2-AG), whose levels are regulated by specific enzymes of biosynthesis and degradation.^9^ CB_2_R is strongly expressed in mouse spermatogonia, which also possess high levels of 2-AG, suggesting the involvement of an autocrine endocannabinoid signal in the mitotic phase of spermatogenesis. Indeed it has been demonstrated that CB_2_R-specific activation in spermatogonia promotes *in vitro* meiotic entry.^11^ Recently, we demonstrated that CB_2_R has a physiological role in regulating spermatogenesis *in vivo* as treatment of young mice with a specific CB_2_R agonist/antagonist causes an alteration of the correct temporal progression of spermatogenesis.^12^ Thus ECS, through CB_2_R, could be a part of regulatory network that controls germ cells’ meiotic entry and progression. In order to extend our knowledge on the pro-meiotic role of CB_2_R, in the current study we investigated the function of this cannabinoid receptor in male and female fetal gonads during a developmental window in which female germ cells enter meiosis but gonocytes are still proliferating, with the additional intent of gaining insights into the role of ECS in oocyte maturation, a function remained unclear. In fact, in humans, some evidence suggests that marijuana can reduce female fertility by altering follicular maturation timing and ovulatory function,^13, 14^ but the molecular mechanisms are still unknown. Here we report that CB_2_R is expressed in fetal male and female germ cells and that its activation by JWH133, a selective CB_2_R agonist, triggers meiosis by increasing the number of SCP3-positive cells and the expression of meiotic genes. We also show that acceleration of meiosis in fetal oocytes causes apoptotic cell death, by increasing the percentage of TUNEL-positive cells and of *γ*-H2AX-positive pachytene/diplotene oocytes, and that *in vivo* exposure of fetal gonads to JWH133 significantly affects postnatal oocyte reserve.

## Results

### Cannabinoid receptors CB_1_ and CB_2_ are expressed in male and female fetal gonads

**Figure 1:**
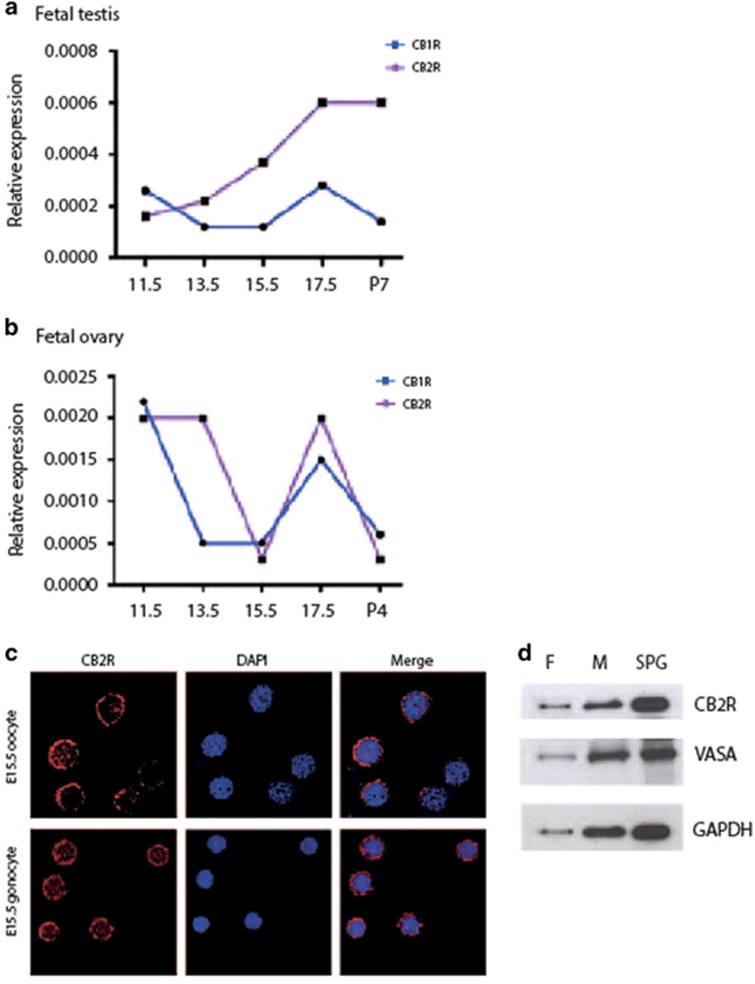
Cannabinoid receptors CB_1_ and CB_2_ are expressed in fetal male and female gonads. (**a**) Relative expression (2^−ΔCt^) of CB_1_R and CB_2_R in male fetal gonad and (**b**) in female fetal gonad at different times of development. (**c**) Immunofluorescence analysis for CB_2_R in both E15.5 male and female fetal germ cells shows the membrane localization of the receptor. (**d**) Western blotting analysis of CB_2_R in isolated E15.5 female (F) and male (M) fetal germ cells. Isolated 7 dpn spermatogonia (SPG) were used as positive control.^12^ VASA is used as a marker of germ cells

The expression of CB_1_ and CB_2_ receptors (CB_1_R and CB_2_R) in male and female fetal gonads at different stages of development from E11.5 to E17.5 was investigated. By qRT-PCR analysis we found that CB_1_R was constantly expressed from E11.5 to postnatal age 7 (dpn) in male gonads (Figure 1a), while CB_2_R expression increased from E11.5 to E17.5 and its expression did not change until 7 dpn. By comparing CB_2_R with CB_1_R mRNA levels, we found that CB_2_R is expressed at higher levels both in fetal and early postnatal development (Figure 1a).

In female gonads, a similar pattern of mRNA expression for both CB_1_R and CB_2_R receptors was observed during fetal development (Figure 1b), and their expression drastically dropped after birth.

Protein expression of CB_2_R and CB_1_R was evaluated by immunofluorescence on disaggregated fetal gonads (Figure 1c). CB_2_R antibody strongly immunostained both purified male and female fetal germ cells at the membrane level. A similar pattern of staining was also observed with anti-CB_1_R antibody (Supplementary Figure S1A), indicating that both the receptors localized on the membrane. By western blotting, we confirmed that CB_2_R was expressed at the protein level both in E13.5 purified male and female germ cells (Figure 1d).

### Activation of CB_2_R signaling promotes gonocyte meiotic entry

**Figure 2:**
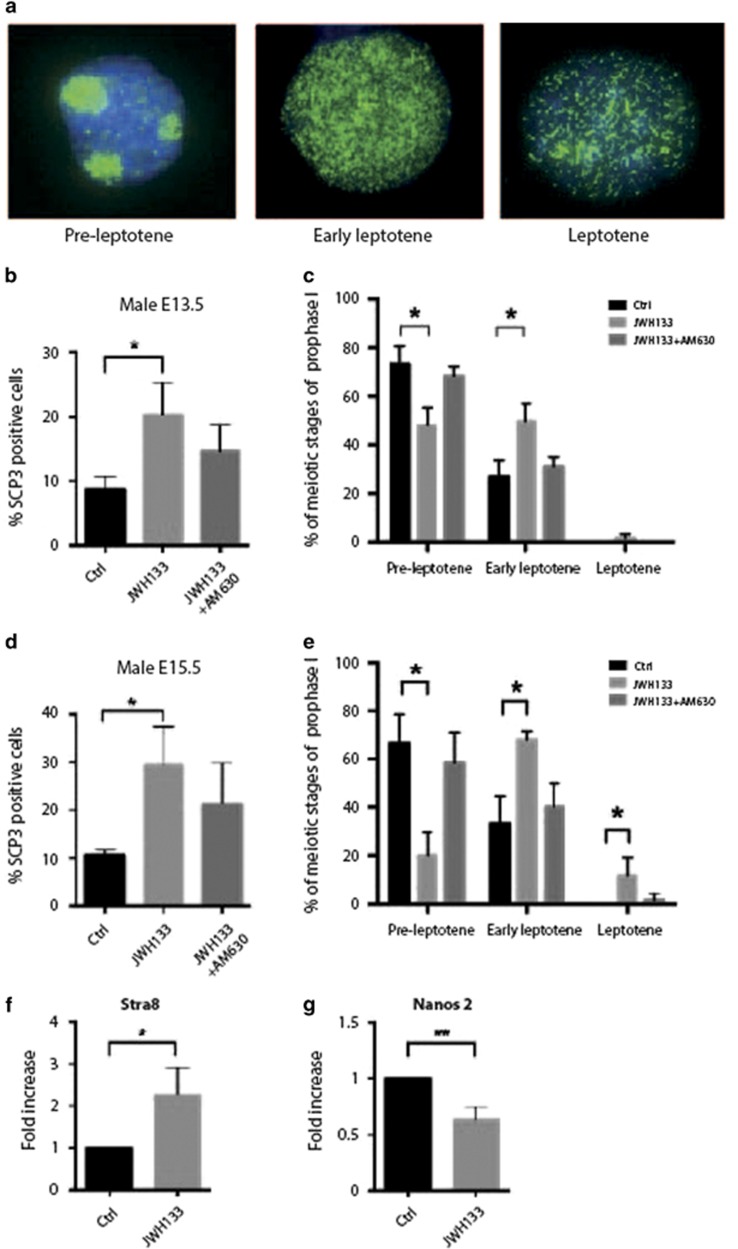
Activation of CB_2_R promotes gonocyte meiotic entry. (**a**) Representative immunofluorescence images showing SCP3 (green) organization on nuclear spreads at the stages of preleptotene, early leptotene and leptotene cells of meiotic prophase I. (**b**) Histogram representing the percentage of nuclei with meiotic SCP3 staining in male germ cells from E13.5 gonads after 48 h of culture in the absence or presence of JWH133 alone or in combination with AM630. (**c**) Percentage of preleptotene, early leptotene and leptotene nuclei in cultures of E13.5 male germ cells treated and untreated for 48 h with JWH133 alone or in combination with AM630. (**d**) Histogram representing the percentage of nuclei with meiotic SCP3 staining in E15.5 male germ cells treated and untreated for 48 h with JWH133 alone or in combination with AM630. (**e**) Percentage of preleptotene, early leptotene and leptotene nuclei in cultures of E15.5 male germ cells treated and untreated for 48 h with JWH133 alone or in combination with AM630. (**f** and **g**) Real-time PCR of meiotic genes *Stra8* and *Nanos2* in E13.5 male germ cells treated or not for 48 h with JWH133. Data were collected from at least three different experiments, using a minimum of 10 embryos for each one. *P<0.05 and **P<0.01

As we already demonstrated that *in vitro* and *in vivo* CB_2_R activation induced meiotic entry and meiotic progression of postnatal spermatogonia, we investigated whether fetal gonocytes stimulation with JWH133 could induce meiotic entry also at the time in which meiosis is prevented. Disaggregated male gonads at E13.5 and E15.5 were cultured for 48 h in the presence of CB_2_R agonist. At the end of the culture, nuclear spreads were performed and SCP3 staining pattern was evaluated as reported in Figure 2a. Our results showed that, at E13.5, stimulation of CB_2_R with JWH133 led to a significant increase in the percentage of SCP3-positive gonocytes (20.3±5.03%) over control untreated cells (8.7±2.08%) (Figure 2b) and this percentage triplicated at E15.5 (JWH133 cells 29.3±8.02% *versus* control cells 10.66±1.15%). In both cases, treatment with the CB_2_R inhibitor, AM630, reversed the JWH133 effects (Figures 2b and d). The enrichment of SCP3-positive gonocytes at E13.5 was essentially due to the increase in early leptotene (50±6.97% of JWH133 treated cells *versus* 26.7±7.23% of control cells) and to a concomitant decrease of preleptotene cells (48.3±7.09% of JWH133-treated cells *versus* 77.3±7.23% of control cells) (Figure 2c). At E15.5, we found a significant decrease of preleptotene in JWH133-treated SCP3-positive gonocytes (20.3±10.5%) over control cells (66.7±11.5%) with a concomitant increase of early leptotene (67.6±4.04%) and leptotene cells (12.1±7.45%) (Figure 2e).

To understand whether meiosis was correlated with the expression of pro-meiotic genes or with repression of antimeiotic genes, by qRT-PCR we tested the mRNA levels of *Stra8* and *Nanos2*, which have been reported to be positively or inversely correlated to meiosis onset, respectively.^6, 15^ We found that, according with its positive role in the meiotic entry, JWH133 significantly upregulated *Stra8* (2.33±0.57) and downregulated *Nanos2* mRNA levels (0.63±0.11) in treated cells compared with control cells (Figure 2f). Interestingly, no differences in the percentage of SCP3-positive cells nor in the distribution of the meiotic stages between treated and untreated cells were detected following stimulation of male fetal gonads with CB_1_R agonist ACEA, indicating that CB_1_R did not have a role in male germ cell differentiation (Supplementary Figures S1B and C).

Our results suggest that CB_2_R acts as a positive regulator of meiotic entry of fetal gonocytes as previously demonstrated for postnatal spermatogonia and suggest that activation of this receptor can force a process that normally is initiated only in the postnatal testis.

### Activation of CB_2_R signaling promotes fetal oocyte meiotic progression

**Figure 3:**
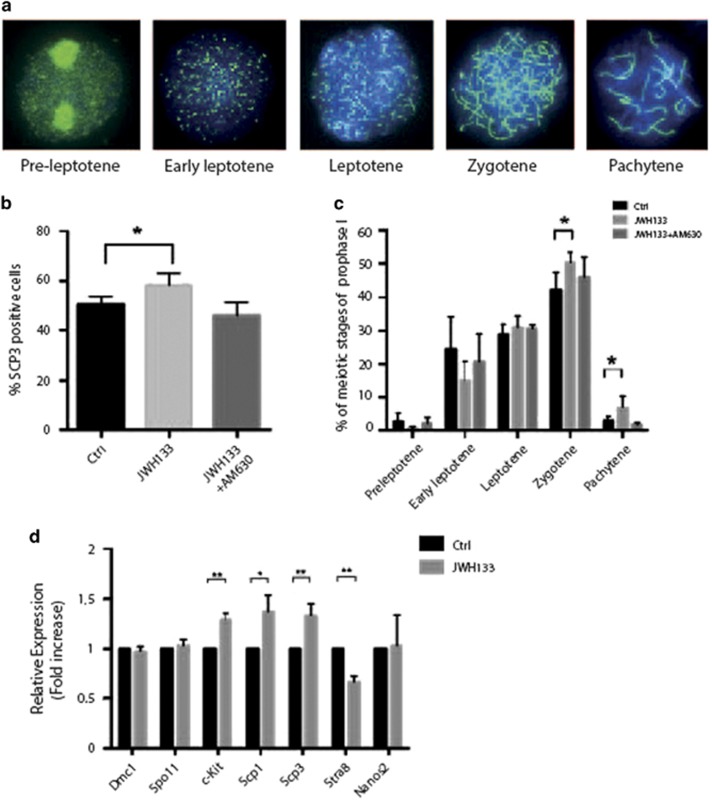
Activation of CB_2_R promotes fetal oocyte meiotic entry at E13.5. (**a**) Representative immunofluorescence images showing SCP3 (green) organization on nuclear spreads at the stages of preleptotene, early leptotene, leptotene, zygotene and pachytene cells of meiotic prophase I. (**b**) Histogram representing the percentage of nuclei with meiotic SCP3 staining in female germ cells from E13.5 gonads treated or not with JWH133 alone or in combination with AM630 for 48 h. (**c**) Percentage of meiotic nuclei at different stages of prophase I in female germ cells from E13.5 gonads treated or not for 48 h with JWH133 alone or in combination with AM630. (**d**) Real-time PCR of meiotic genes *Dmc1*, *Spo11*, *c-Kit*, *SCP1*, *SCP3*, *Stra8* and *Nanos2* in female germ cells from E13.5 gonads treated or not for 48 h with JWH133. Data were collected from at least three different experiments, using a minimum of 10 embryos for each one. *P<0.05 and **P<0.01

**Figure 4:**
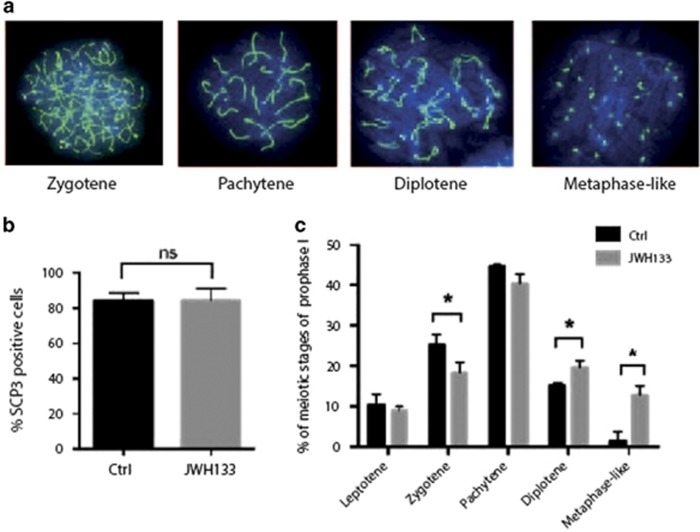
Activation of CB_2_R promotes fetal oocyte meiotic progression at E15.5. (**a**) Representative immunofluorescence images showing SCP3 (green) organization on nuclear spreads at the stages of leptotene, zygotene, pachytene, diplotene and metaphase-like cells of meiotic prophase I. (**b**) Histogram representing the percentage of nuclei with meiotic SCP3 staining in female germ cells from E15.5 gonads treated or not with JWH133 for 48 h. (**c**) Percentage of meiotic nuclei at different stages of prophase I of female germ cells from E15.5 gonads treated or not for 48 h with JWH133. Data were collected from at least three different experiments, using a minimum of 10 embryos for each one. NS, not significant. *P<0.05

During fetal development, female primordial germ cells (PGCs) asynchronously enter meiotic prophase I at E12.5.^16^ We then asked whether CB_2_R stimulation *in vitro* might influence meiotic entry and/or progression of fetal female germ cells obtained from E13.5 and E15.5 ovaries. Disaggregated E13.5 ovaries were treated with JWH133 for 48 h and then nuclear spreads were analyzed for SCP3 staining (Figure 3a). Stimulation with the CB_2_R agonist determined a small but significant increase of total SCP3-positive cells (58.5±4.6% *versus* 50.6±3.1% of control cells), and this effect was completely abolished by the treatment with the CB_2_R antagonist AM630, suggesting the specific involvement of the receptor in promoting this effect (Figure 3b). A more detailed analysis of meiotic stages showed a significant increase of zygotene (50.2±3.4% *versus* 42.3±5.18% of control cells) and pachytene cells (6.0±2.2% *versus* 2.75±1.26% of control cells) following JWH133 treatment, concomitantly with a small decrease in preleptotene and early leptotene stages, while the percentage of leptotene was unchanged (Figure 3c). The effect was reverted by the addition of AM630. This result suggested that activation of CB_2_R in E13.5 oocytes accelerated meiotic progression up to the pachytene stage. To validate our morphological observation, we performed qRT-PCR for the expression of pro-meiotic and meiotic genes. Expression of *c-Kit*, *Scp1* and *Scp3* was upregulated in JWH133-treated oocytes, while *Dmc1* levels did not change (Figure 3d). JWH133 treatment did not affect *Nanos2* levels (which were almost undetectable) (Figure 3d). On the contrary, the expression of *Stra8*, which is required for meiotic initiation at E12.5 but that rapidly declines at around E16.5,^17^ resulted downregulated in stimulated oocytes, confirming that JWH133 accelerated meiotic progression in these cells. To better highlight the positive effect on meiotic progression, we collected fetal ovaries at more advanced developmental age. E15.5 disaggregated ovaries were cultured for 48 h in the presence or not of JWH133 and nuclear spreads were prepared and stained with anti-SCP3 antibody to identify meiotic figures. As expected, meiotic figures in control cells at E15.5 were at more advanced meiotic stages with respect to those identified at E13.5 (Figure 4a). We found that JWH133 treatment did not affect the percentage of total SCP3-positive cells (84.7±6.5%) compared with the control group (83.3±4.04%) (Figure 4b), but interestingly, the relative percentage of the meiotic stages was modified. In particular, JWH133 treatment induced a decrease in the percentage of zygotene cells (18.3±2.5% *versus* 25.9±3.61% of control cells) concomitantly with an increase of diplotene (19.7±1.53% *versus* 15.3±0.6% of control cells) and the appearance of metaphase-like cells (12.7±2.51% *versus* 1.3±2.4% of control cells) (Figure 4c), further suggesting that CB_2_R stimulation promoted meiotic progression of fetal oocytes. As specific phosphorylation of histone H3 at Ser10 has been tightly coupled to chromatin condensation during mitosis and meiosis and metaphase chromosomes are always found to be heavily phosphorylated by Cdk1,^18^ we checked for H3 pSer10 positivity in metaphase-like chromosomes from the nuclear spreads of JWH133-treated E15.5 oocytes. Surprisingly, we found no p-H3 staining on metaphase-like chromosomes, indicating that chromosome condensation was not mediated by Cdk1 activation (Supplementary Figure S2A). A positive control of H3 pSer10 positivity in isolated spermatocytes treated with okadaic acid is shown in Supplementary Figure S1B.

### Activation of CB_2_R induces *γ*-H2AX foci and apoptosis in cultured fetal oocytes

**Figure 5:**
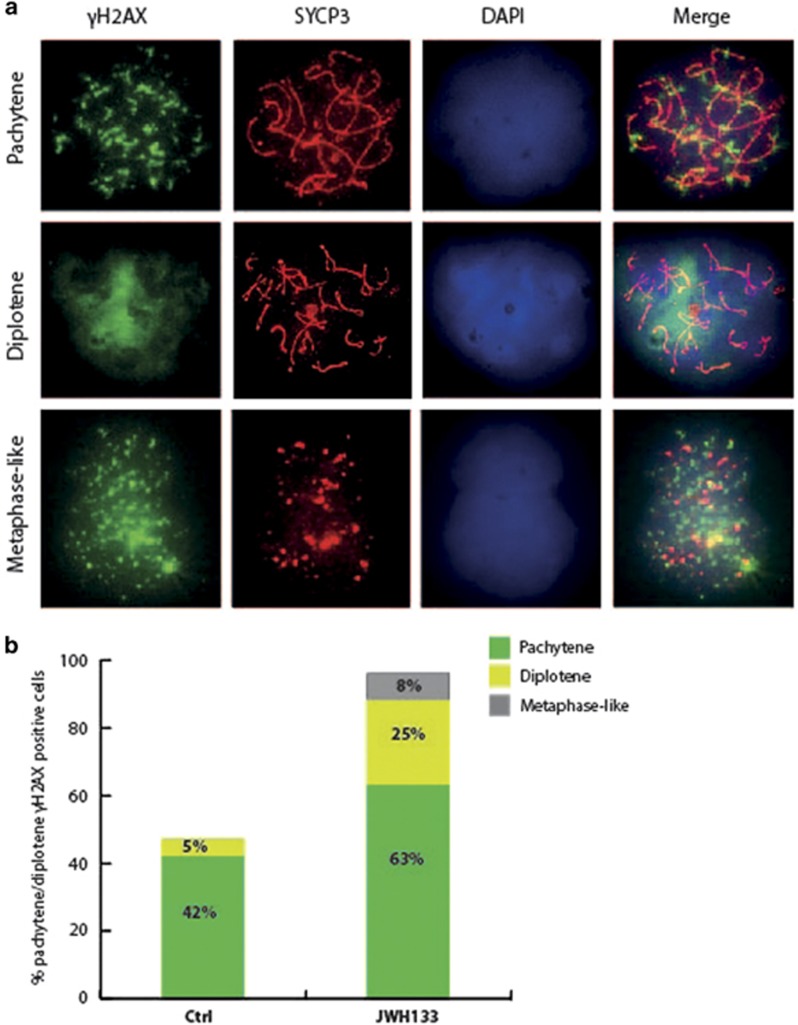
Activation of CB_2_R increases *γ*-H2AX foci in fetal oocytes. (**a**) Representative immunofluorescence images showing staining of SCP3 (red) and *γ*-H2AX (green) on pachytene, diplotene and metaphase-like cells from E15.5 ovary. (**b**) Percentage of double-positive SCP3 and *γ*-H2AX oocytes at the stages of pachytene, diplotene and metaphase-like from E15.5 ovary, treated or not with JWH133 for 48 h. Data were collected from at least three different experiments, using a minimum of 10 embryos for each one

**Figure 6:**
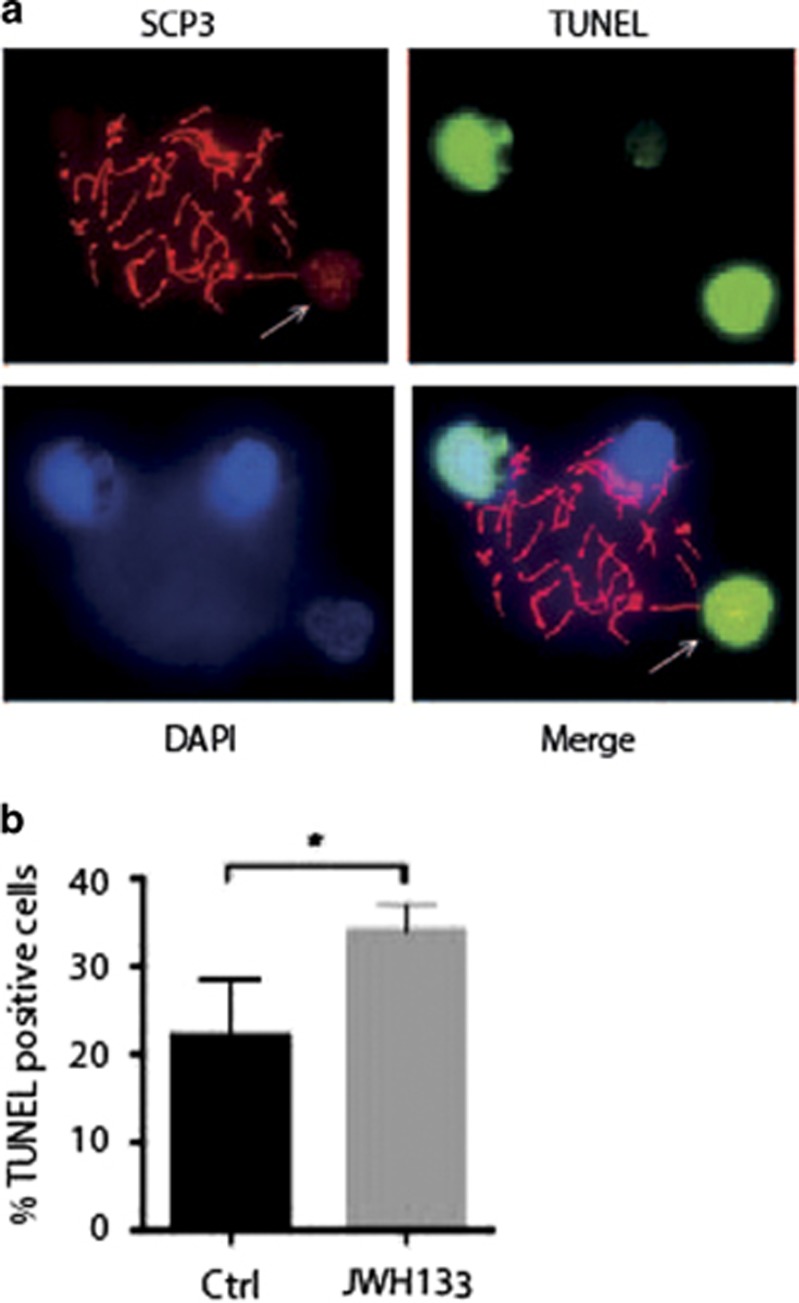
Activation of CB_2_R increases the number of terminal deoxinucleotidyl transferase-mediated dUTP-fluorescein nick end labeling (TUNEL)-positive oocytes. (**a**) Representative image of spreads nuclei of E15.5 female germ cells stained for SCP3 (red) and TUNEL (green). An SCP3-positive cell at the pachytene stage, a TUNEL-positive cell with disassembled pattern of synaptonemal complex (indicated by white arrow) and TUNEL-negative cells are shown. (**b**) Percentage of SCP3 and TUNEL double positive in female germ cells from E15.5 gonads treated or not with JWH133 for 48 h. Data were collected from at least three different experiments, using a minimum of 10 embryos for each one. *P<0.05

We next evaluated DNA integrity and chromatin organization in JWH133-treated fetal oocytes by staining with *γ*-H2AX. In meiosis, H2AX phosphorylation (*γ*-H2AX) is detected predominantly during the leptotene stage when double-strand breaks (DSBs) occur and it has a role in the recruitment of DNA repair factors and DNA damage-signaling proteins. Although *γ*-H2AX staining is lost during meiotic progression, it is retained up to pachytene stage in the regions of chromosome asynapsis.^19, 20, 21^ To verify whether activation of CB_2_R caused accumulation of oocytes with asynapsed DNA at pachytene/diplotene stages, we performed a double staining with SCP3 and *γ*-H2AX antibodies to count *γ*-H2AX-reactive foci only in the pachytene and diplotene nuclei (Figure 5a). We found that, after 48 h of culture, 42 and 5% of E15.5 control oocytes displayed *γ*-H2AX spots at the pachytene and diplotene stages, respectively. JWH133 treatment caused a significant increase of *γ*-H2AX-positive nuclei at pachytene stage (63%), diplotene (25%) and metaphase cells (8% *versus* 0% in control), (Figure 5b), indicating that drug treatment caused an enrichment of DNA-damaged cells. To investigate whether JWH133 treatment induced apoptosis in treated oocytes, we performed a TUNEL assay and SCP3 staining on JWH133-treated E15.5 oocytes. We found that the number of SCP3/TUNEL double-positive oocytes was increased after the treatment (Figures 6a and b), suggesting that meiosis acceleration induced by JWH133 was not followed by correct DNA repair, thus increasing oocyte apoptosis rate.

### Ovarian reserve is altered following in utero exposure to JWH133

**Figure 7:**
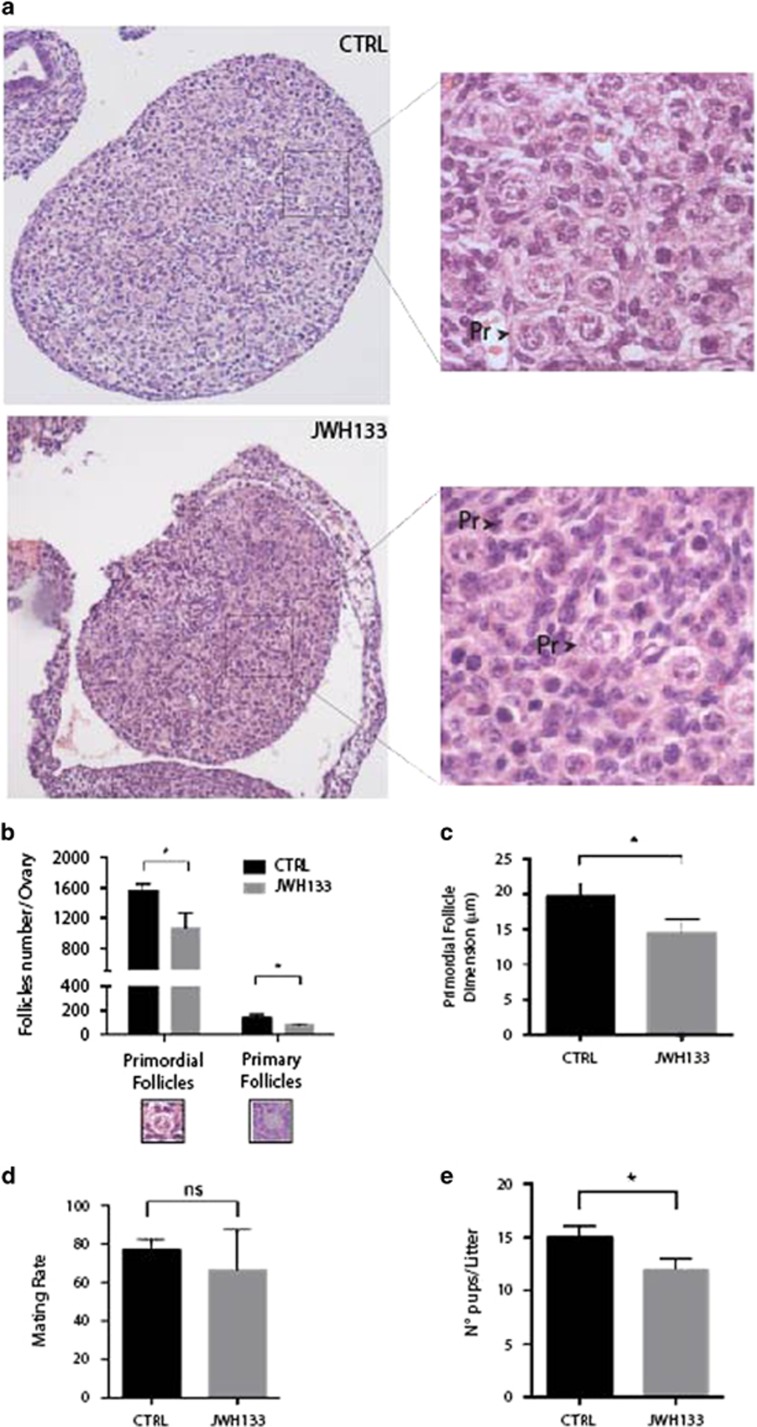
*In vivo* treatment of pregnant mice with JWH133 affect ovarian reserve of female pups. (**a**) Morphological staining with H&E of 1 dpn ovaries *in utero* exposed to JWH133 shows a reduction in follicle number and size as shown at higher magnification on the right (Pr.: primordial follicles). (**b**) The histograms show a significant reduction in primordial and primary follicle number in the ovary of F1 offspring *in utero* exposed to JWH133 with respect to vehicle-exposed offspring. (**c**) A significant reduction in the diameter of primordial follicles was detected in the ovaries of F1 from JWH133-treated pregnant mice with respect to F1 primordial follicles from control pregnant mice. (**d**) Mating rate of F1 female from JWH133-treated pregnant mice was unchanged with respect to mating rate of F1 control mice. (**e**) Litter size of F2 from JWH133 F1 female was significantly reduced with respect to that F1 control mice. Data were collected using a minimum of three 1 dpn female pups. NS, not significant. *P<0.05

To evaluate whether CB_2_R activation could affect the fetal germ cell development *in vivo*, we treated pregnant females intraperitoneally with JWH133 (0,3 mg JWH133/kg) at E12.5 for 4 consecutive days (Figure 7a). At 1 dpn, the weight of pups obtained from control and treated pregnant mice was analyzed. As shown in Supplementary Figure S3, all the JWH133 newborn litters, either male or female pups, showed a significant decreased body weight with respect to sham-treated control litters. Ovaries (Figure 7a) and testes (Supplementary Figure S4) of the newborns (F_1_) were collected and analyzed at morphological level by staining with H&E. At this age, the mouse ovary typically contains germ cell cysts, primordial follicles in which oocytes are surrounded by flattened pregranulosa cells and very few primary follicles in which granulosa cells take on a cuboid shape. By counting the number of oocytes enclosed in follicles in each histological section (see Materials and Methods), we found that the number of primordial, as well as primary follicles, was significantly reduced in the ovaries of JWH133 newborns in comparison with the ovaries from sham-treated newborns (Figure 7b). Furthermore, the diameter of primordial follicle was significantly smaller in JWH133 ovaries (Figure 7c). On the contrary, morphological analysis of the testes showed no differences in JWH133 treated compared with control male pups, and gonadal architecture displayed regular tubular structure containing Sertoli cells and central gonocytes (Supplementary Figure S4) in both samples.

Considering the reduced number of follicles in the ovaries of F_1_ females, they were crossed with untreated males and their reproductive performance was analyzed by monitoring their mating rate and the number of pups delivered at F_2_. No differences in the mating rates were detected between JWH133 F_1_ females and relative controls (Figure 7d), but interestingly, we observed a significant decrease of the F_2_ litter size (12±1.00 pups from JWH133-treated F_1_ females with respect to 15±1.00 pups from control females, Figure 7f). These results indicated that exposure of pregnant mice to a specific CB_2_R agonist affected newborn growth and, most importantly, reduced the ovarian reserve and the reproductive capacity of female pups of the first generation.

## Discussion

Meiosis is a critical phase during gametogenesis and the molecular mechanisms that regulate this process in mammals are not fully understood. We previously demonstrated that cannabinoid receptor CB_2_ regulated meiotic entry of postnatal spermatogonia *in vitro* and *in vivo*, and its activation induced an acceleration of the onset of spermatogenesis that disrupted the temporal dynamics of the spermatogenic cycle.^12^ To understand whether CB_2_R activation could induce and/or accelerate meiosis also in male and female fetal germ cells, JWH133, a selective agonist, recommended for the studies on the role of CB_2_R in biological processes,^22^ was used. In this study, we present evidences that CB_2_R has an important role during fetal life in (1) inducing male meiosis, in (2) inducing and accelerating female meiosis and in (3) decreasing the pool of primordial and primary follicles, negatively impacting on the ovarian reserve in the offspring.

Meiosis is prevented in gonocytes during fetal life by the presence of the retinoid-degrading enzyme CYP26B1,^3, 6^ which decreases retinoic acid (RA) levels within the gonad, thus inhibiting *Stra8* and activating *Nanos2* expression.^3, 15^ This condition is reverted after birth and local increase of RA concentrations induces meiotic entry of differentiating spermatogonia.^7^ Pharmacological stimulation of CB_2_R with JWH133 activated the meiotic program in fetal male germ cells by increasing *Stra8* gene expression and by decreasing *Nanos2* expression, similarly to what we previously observed in postnatal spermatogonia following JWH133 or RA stimulation.^12^ The increase of SCP3-positive cells induced by the drug included early leptotene and few leptotene spermatocytes but not more advanced stages, indicating that activation of CB_2_R signaling only promoted meiotic entry of gonocytes, but it did not support the progression of meiosis. A similar effect has been observed on postnatal spermatogonia that did not proceed beyond the zygotene stage following *in vitro* JWH133 stimulation.^12^ These results suggest that CB_2_R facilitates entry and progression only throughout the early stages of meiosis both in fetal and postnatal male germ cells; however, they do not acquire the competence to overcome meiotic checkpoints to proceed toward the end of prophase I. One possible explanation is the lack of all the molecular factors required for meiotic progression in meiosis-incompetent gonocytes/spermatogonia. For example, it has been suggested that transcripts produced at the onset of meiosis would require specific stabilization to perform their functions throughout the prolonged phases of prophase I^23^ and these factors could be not induced by drug treatment.

**Figure 8:**
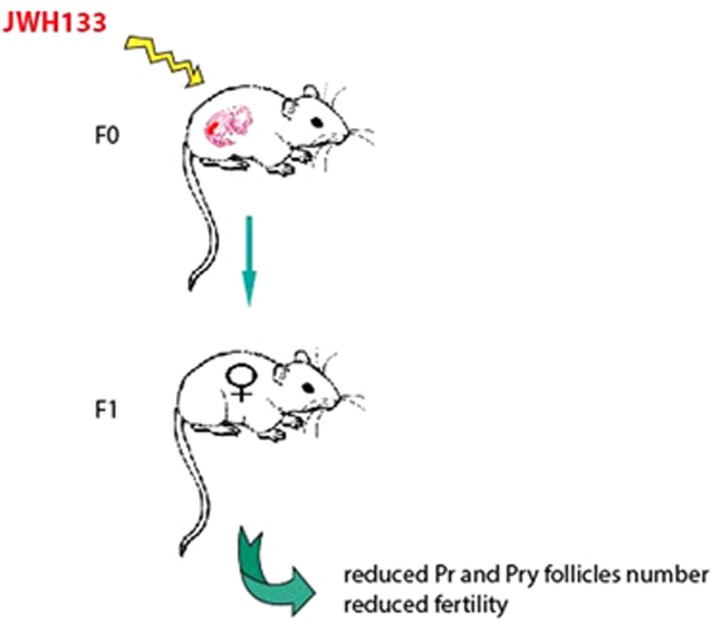
Schematic representation of the effects of CB_2_R overactivation on fetal ovary. Pregnant females were intraperitoneally injected with JWH133 for 4 consecutive days at the onset of meiosis in oocytes (E12.5). Morphological analysis of F1 ovary at birth (1 dpn) showed a reduced number of primordial (Pr) and primary (Pry) follicles with respect to control. F1 JWH133 female crossed with untreated male showed a decreased fertility with a smaller litter size at F2

Few information are available about CB_2_R expression and function in female germ cells. Evidences suggested that both CB_1_R and CB_2_R are expressed in human oocytes,^24^ but their role is actually unknown. We demonstrated that CB_2_R is expressed in mouse fetal oocytes, starting from E11.5 throughout all the female fetal life. By using JWH133, we showed that CB_2_R has a role in promoting meiotic progression of E13.5 oocytes, as demonstrated by the enrichment of SCP3-positive cells at the zygotene and pachytene stages. Moreover, we found upregulation of pro-meiotic (*c-kit*) and meiotic genes (*Scp1* and *Scp3*) and downregulation of *Stra8* gene expression, further confirming at molecular level that treated oocytes were progressing through meiosis. The same treatment on more advanced meiotic oocytes, at E15.5, confirmed that JWH133 accelerated meiotic progression up to a stage that resembled metaphase I meiosis. We named these meiotic figures as metaphase-like cells for the lack of co-staining with the phospho-histone H3, a specific marker of metaphase chromosomes.^18^ We hypothesized that correct chromatin condensation was not occurring in JWH133-treated oocytes due to retarded DSB repair, thus inducing chromosome asynapsis. This hypothesis was confirmed by the observation that a significantly higher percentage of *γ*-H2AX-positive pachytene and diplotene cells was found in JWH133-treated compared with control oocytes. The increase of *γ*-H2AX-positive cells at late meiotic stages and the presence of metaphase-like nuclei was paralleled by an increase of TUNEL-positive nuclei in the treated group, suggesting that CB_2_R activation promoted meiotic chromatin condensation without DSB repair, and this condition was followed by apoptotic cell death. This effect was relevant *in vivo*. *In utero* CB_2_R agonist treatment significantly affected the development of oocytes in female offspring, as indicated by the significant decrease in the number and the size of primordial and primary follicles in female newborns from JWH133 pregnant females. The reduced ovarian reserve of F_1_ female was maintained during adult life and determined reduced fertility. On the contrary, we did not find any germ cell defects in male pups from JWH133 pregnant females. As CB_2_R *in vivo* stimulation was performed in a developmental window in which male germ cells still divide, it is possible that cell proliferation was balancing cell death following inappropriate meiotic entry. Altogether our findings highlight a pro-meiotic role of CB_2_R in female reproduction never documented previously and indicate that interfering with endogenous function of CB_2_R in germ cells during fetal life could have adverse effects on the fertility and on the establishment of ovarian reserve of the F_1_ generation as proposed in Figure 8. Females that enter puberty with a small ovarian reserve are at risk of a shorter reproductive lifespan, as their oocyte reserve is expected to be depleted faster.

In conclusion, this study defines the importance of the endocannabinoids and the cannabinoid CB_2_R for germ cell development during fetal life. The potential clinical relevance of our observations is that the consumption of marijuana or the administration of cannabinoid‐based drugs during pregnancy might interfere with ECS in germ cells of the developing fetus thus impairing female fertility and ovarian reserve of F_1_ generation. Future studies should be focused to investigate the molecular mechanism of these alterations to understand if they are maintained also in the future generations.

## Materials and methods

### Animals

All albino *Swiss CD1* female mice (*Mus musculus*) at 6–8 weeks of age were caged with males at a ratio of 1 : 1 overnight and checked for a vaginal plug the following morning. The presence of a vaginal plug was considered 0.5 dpc (days *post coitum*, also referred as embryonic day (E)). The day in which the pups were born was considered 1 dpn. All mice were housed under 16/8 h light/dark cycles at 25 °C with access to food and water *ad libitum*. Mice were killed at different embryonic ages, in accordance with European Community guidelines. Experimental protocols were performed in accordance with guidelines established by the European Legislation (Directive 2010/63/EU) and approved by University of Rome Tor Vergata IACUC and by Ministry of Health (legal authorization N. 1028/2015-PR).

### Isolation and culture of fetal germ cells and spermatocytes

Ovaries and testes from CD1 mouse embryos were separated by microdissection from the mesonephros in prechilled PBS (10 mM, pH 7.4) under a stereomicroscope. The collected gonads were digested with trypsin and DNAseI (Sigma-Aldrich, MO, USA) and then cultured in six-well culture dishes with DMEM (Sigma-Aldrich) supplemented with 10% FBS (Gibco, CA, USA) to promote adhesion of somatic cells at 37 °C in a 5% CO_2_.^15^ Both male and female disaggregates were treated for 48 h in the presence or absence of 1 *μ*M JWH133 (Tocris, Bristol, UK). To purify E13.5 male and female germ cells, disaggregated gonadal cells were resuspended in a 1 : 100 of TG-1 antibody solution in PBS–BSA 1% and subjected to MiniMACSs purification onto IgM-conjugated beads (Miltenyi Biotec, Cologne, Germany).^25^ Purified TG-1-positive germ cells were analyzed for western blotting or immunofluorescence analysis.

Spermatocytes were purified from adult testes as previously described.^11^ Cells were cultured at 32 °C for 4 h in the presence or absence of 0.5 *μ*M okadaic acid (OA) (Calbiochem biotec, CA, USA).

### Western blotting

Total protein extraction from purified PGCs was performed as previously described.^10^ Western blotting analysis was carried out using the following primary antibody: rabbit anti-CB2 (101550; Cayman Chemical, Ann Arbor, MI, USA), rabbit anti-VASA (13840; Abcam, Cambridge, UK) and rabbit anti-actin (a2066; Sigma-Aldrich).

### Chromosome spreads and immunofluorescence staining

Nuclear spreads were performed as previously described.^11^ SCP3 antibody was used to identify the chromosomal axial elements at prophase I of meiosis. Slides were incubated with 1 : 200 anti- SCP3 (Santa Cruz, TX, USA) in blocking solution (10% goat serum, 3% BSA, 0.05% Triton X-100 in PBS), overnight at 4 °C. Phosphorylated-histone H3 (pH3) was used to verify the presence of metaphase I stage (Santa Cruz, 1 : 200 in blocking solution). Anti-*γ*-H2AX antibody (Upstate, 1 : 100 in blocking solution) was used to detect the asynapsed chromosome regions. Secondary antibody (Alexa Fluor 488 or 594, 1 : 200 in blocking solution) was incubated 1 h at 37 °C.

For immunofluorescence analysis, cells were incubated with 1 : 100 anti-CB_1_R and anti-CB_2_R (Cayman Chemical) in PBS–BSA 0.5% overnight at 4 °C. Secondary antibody (Alexa Fluor 594) 1 : 200 was added and incubated 1 h at 37 °C in PBS–BSA 0.1%.

All spreads and immunofluorescence slides were counterstained with DAPI and were viewed with Leica CTR 6000 microscope (Wetzlar, Germany).

### TUNEL assay

After the chromosome spreads, TUNEL assay (*In Situ* Cell Death Kit, Roche Diagnostics, Indianapolis, IN, USA) was used to detect the apoptotic nuclei. TUNEL reaction mixture was prepared by adding the total volume (5 *μ*l) of enzyme solution to the remaining 45 *μ*l of label solution to obtain 50 *μ*l TUNEL reaction mixtures and incubated with each samples at 37 °C in the dark for 60 min.

### RT-PCR and quantitative real-time PCR

Total RNA was extracted from male and female PGCs cells using Trizol Reagent (Invitrogen, CA, USA) according to the manufacturer’s instructions and 1 *μ*g was used for retrotranscription (RT) using M-MLV reverse transcriptase (Invitrogen). cDNA was used as template for qRT-PCR using SSOADV Universal SYBR Green (Bio-Rad, CA, USA) in a StepOne Plus real time PCR system (Applied Biosystems, CA, USA). The sequences of primers used for qRT-PCR are listed in Supplementary Table S1.

### *In vivo* treatment and histological analysis

To test the effect of the drugs on gonadal development, pregnant mice from E12.5 to E16.5 were intraperitoneally injected with 0.3 mg/kg of JWH133 (Tocris, Bristol, UK) (26). At the end of this period, they were killed and male and female gonads of pups at 0.5 dpn were analyzed at morphological level. Male and female gonads were fixed in Bouin’s solution for H&E staining. Newborns’ testes and ovaries were embedded in paraplast and sectioned at 5 *μ*m on Leica-RM 2035 Microtome (Wetzlar, Germany). Follicles in each ovary were counted serially in every third section through the entire ovary. Only healthy, non-atretic follicles with visible oocyte nuclei were scored.

### Statistical analysis

Continuous variables were summarized as means±S.D. A significance value threshold of 0.05 was used for the current analysis. Student’s *t*-test was used to test for differences between two independent groups, whereas one-way ANOVA followed by Bonferroni test was used for differences among three or more independent groups. All statistical tests were carried out using the GraphPad Prism statistical analysis software package, version 6.0 (GraphPad Software, CA, USA).

## Publisher’s Note:

Springer Nature remains neutral with regard to jurisdictional claims in published maps and institutional affiliations.
