# Supplementary material for: Overactive type 2 cannabinoid receptor induces meiosis in fetal gonads and impairs ovarian reserve
Source: Cell Death Dis. 2017 Oct 5;8(10):e3085–. doi: 10.1038/cddis.2017.496 (PMC5682662; doi:10.1038/cddis.2017.496)
Supplement: Supplementary Figure Legends [file cddis2017496x1.docx]

**Supplemental Information**

**Figure S1. Expression and stimulation of CB_1_ receptor in E13.5 male and female fetal gonads.** (A) The staining for CB_1_ receptor (red) shows an uniform expression at membrane level of the protein in both male and female germ cells at E13.5. (B) Spreads on E13.5 gonocytes show that CB_1_R stimulation with its specific agonist (ACEA) in combination or not with the inhibitor (AM251) does not lead to an increase in SCP3 positive nuclei. (C) The histogram shows that activation and/or inhibition of CB_1_R does not accelerate meiotic progression into prophase I.

**Figure S2. Abnormal chromatin condensation of E15.5 female germ cells at metaphase I stage.** (A) Nuclear spreads of metaphase-like cells stained with SCP3 (green) and with metaphase marker pH3 (red). No pH3 staining was detected in fetal female germ cells from E15.5 gonads treated or not with JWH133. (B) Okadaic acid-treated spermatocytes, used as positive control, stained with both SCP3 and pH3 antibody.

**Figure S3**. JWH133 injection of female pregnant mice causes a reduction of offspring body size. A) The table reports the body weight of F1 offspring at 1, 4 and 10 dpn. B) Representative picture of F1 offspring from JWH133 treated pregnant female and from control pregnant female.

**Figure S4. *In vivo* treatment of pregnant mice with JWH133 does not affect testis development of newborns.** Morphological staining with H&E of 1 dpn testis *in utero* exposed to JWH133 shows no defects within the tubules respect to control counterpart. Data were collected using a minimum of three 1 dpn male pups.


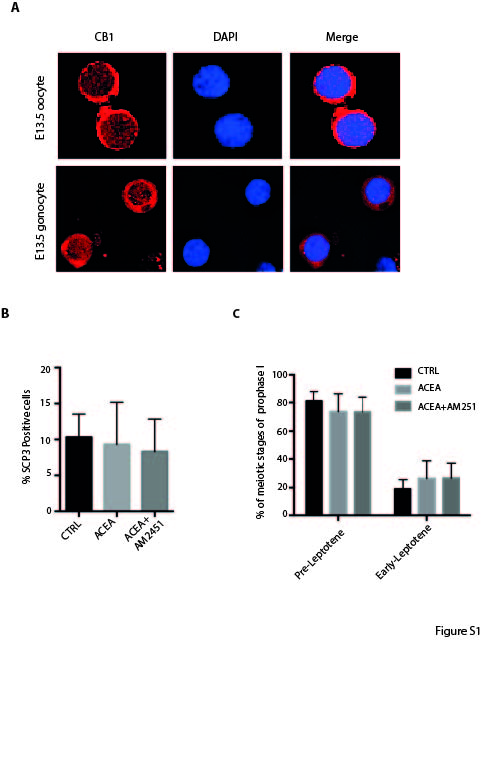


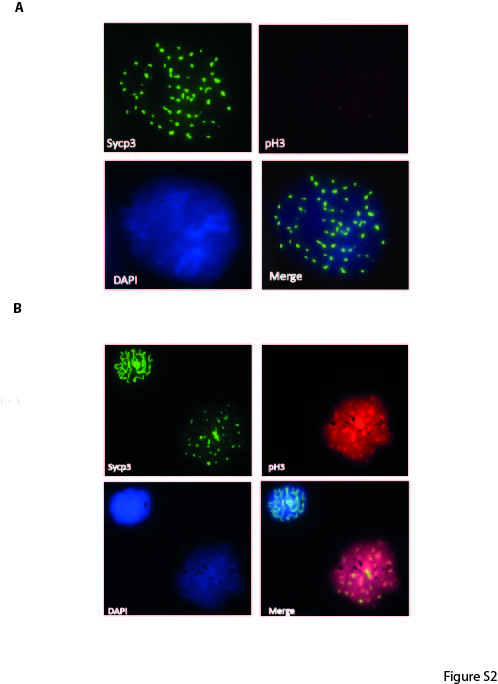


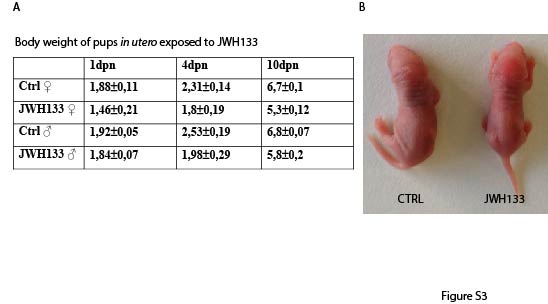


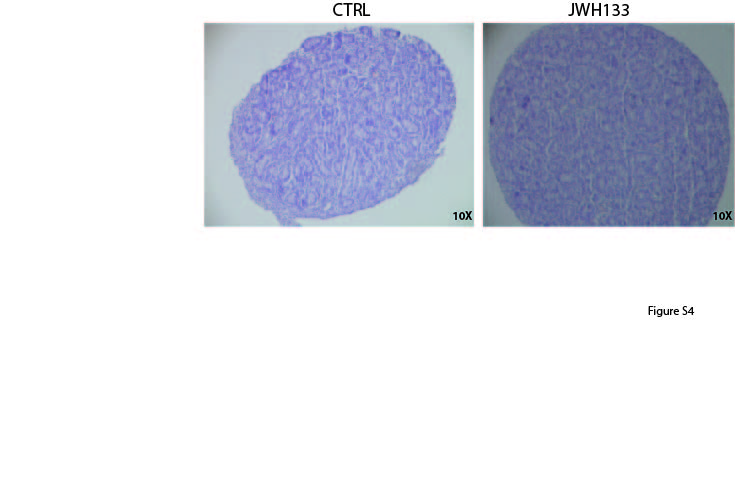


Table S1: Primers sequences

| **Gene** | **Sequences 5'-3'** |
| --- | --- |
| *CB1* | Fw: -CCAAGAAAAGATGACGGCAG  Rv: -AGGATGACACATAGCACCAG |
| *CB2* | Fw: -TCGCTTACATCCTTCAGACAG  Rv: -TCTTCCCTCCCAACTCCTTC |
| *Stra8* | Fw: -GTTTCCTGCGTGTTCCACAAG  Rv: -CACCCGAGGCTCAAGCTTC |
| *Nanos2* | Fw: -AGTGCCATGGACCTACCGCCCTTT  Rv: -TCTCAATTATCGCTTGACTCTGC |
| *c-Kit* | Fw: -GAGACGTGACTCCTGCCATC  Rv: -TCATTCCTGATGTCTCTGGC |
| *Dmc1* | Fw: -CCCTCTGTGTGACAGCTCAAC  Rv: -GGTCAGAATGTCCCGAAG |
| *Scp3* | Fw: -GGAGCTGACATCAACAAAGC  Rv: -GTATATCCAGTTCCCACTGC |
| *Scp1* | Fw: -GCTTCCAGGAGGTTCTGAGG  Rv: -CTCCTGGGCCGTTGTCAG |
| *Spo11* | Fw: -GAAGTGCCTGCCTTCACAAT  Rv: -GCCGACAGAATCATCAAACAT |
| *Actin* | Fw: -CTGTCGAGTCGCGTCCAC  Rv: -GCTTTGCACATGCCGGAG |
