# Supplementary figures and images for: Overactive type 2 cannabinoid receptor induces meiosis in fetal gonads and impairs ovarian reserve
Source: Cell Death Dis. 2017 Oct 5;8(10):e3085–. doi: 10.1038/cddis.2017.496 (PMC5682662; doi:10.1038/cddis.2017.496)

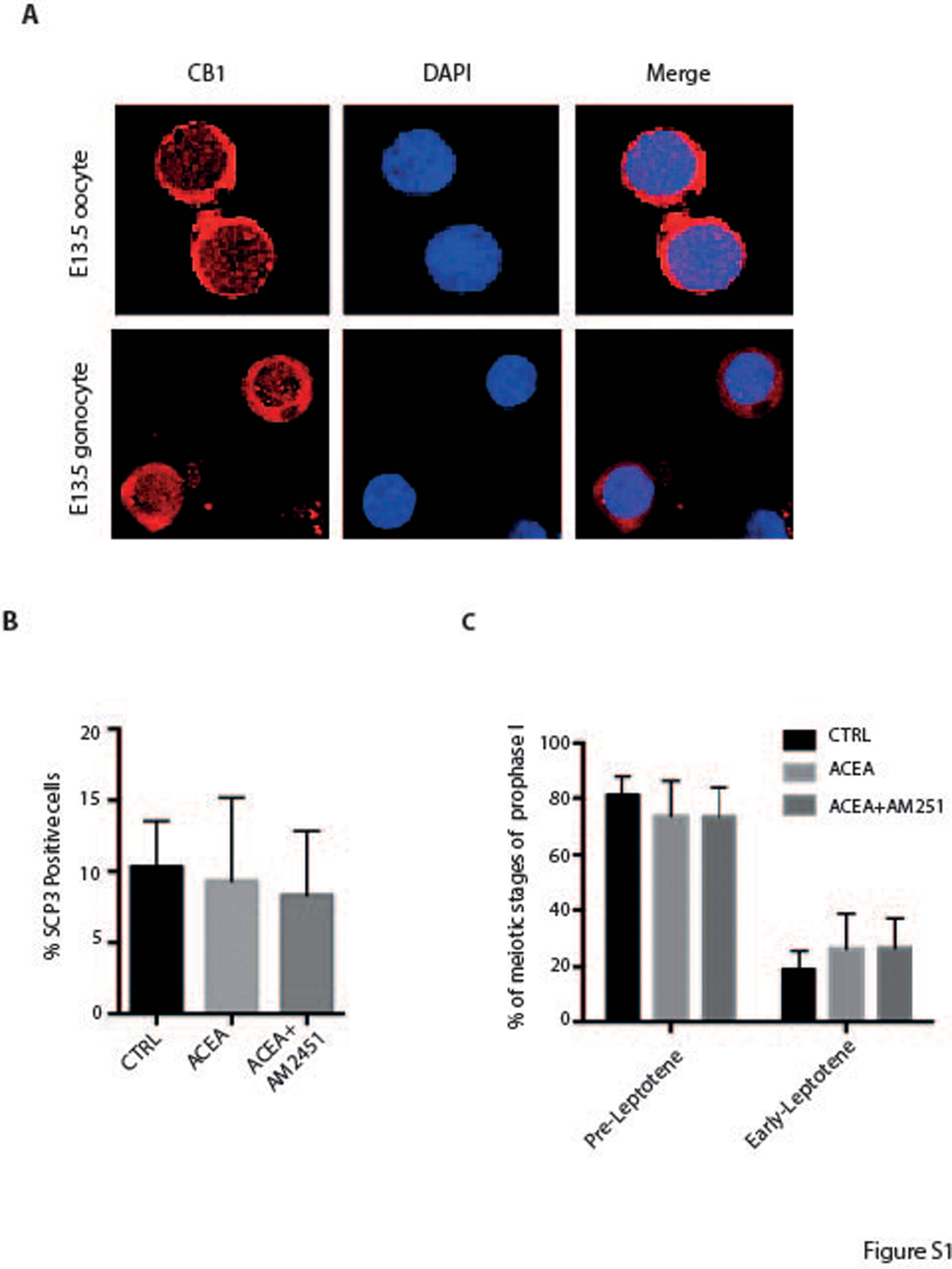

Supplement: Supplementary Figure S1 [file cddis2017496x2.tif]

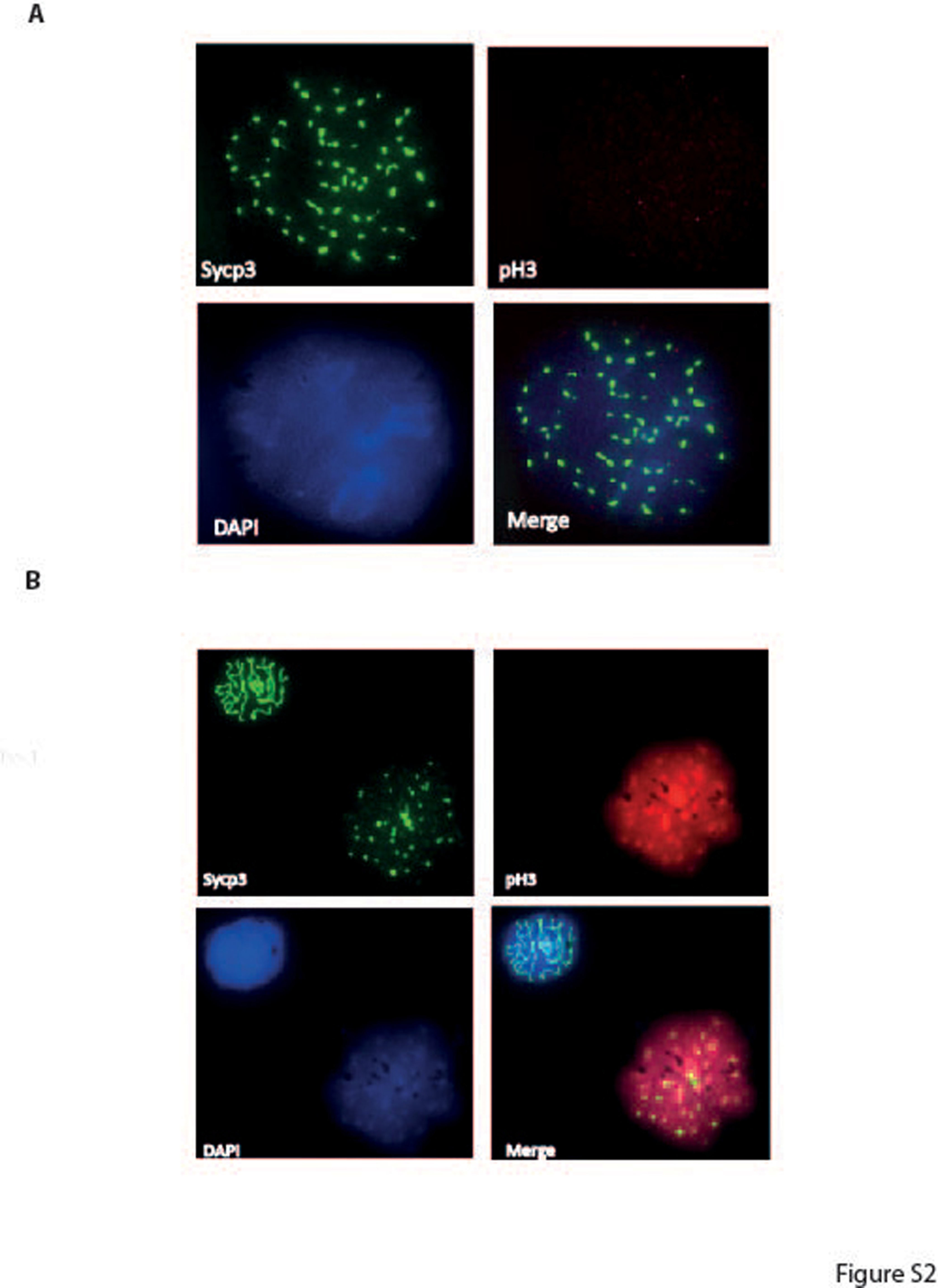

Supplement: Supplementary Figure S2 [file cddis2017496x3.tif]

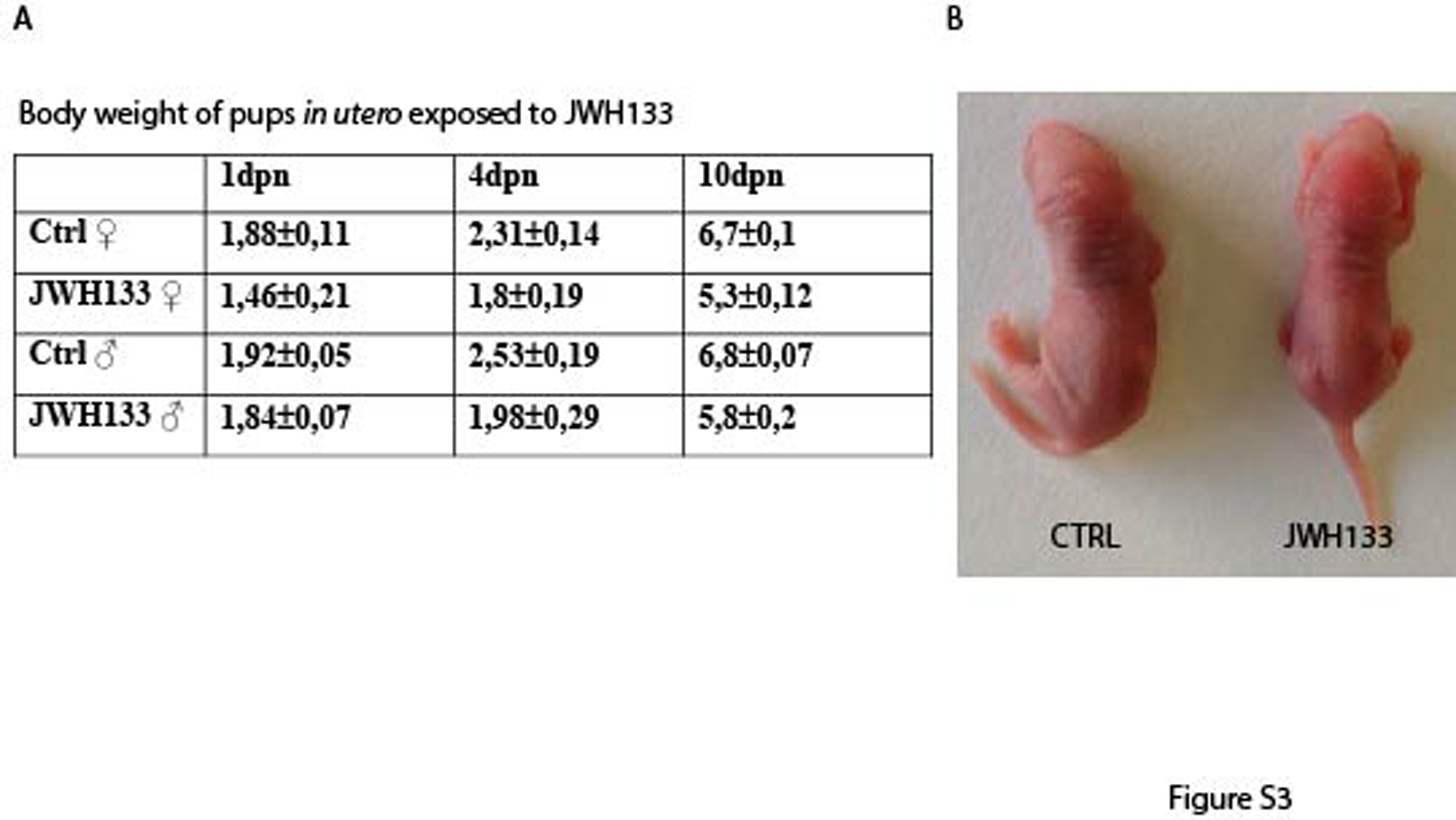

Supplement: Supplementary Figure S3 [file cddis2017496x4.tif]

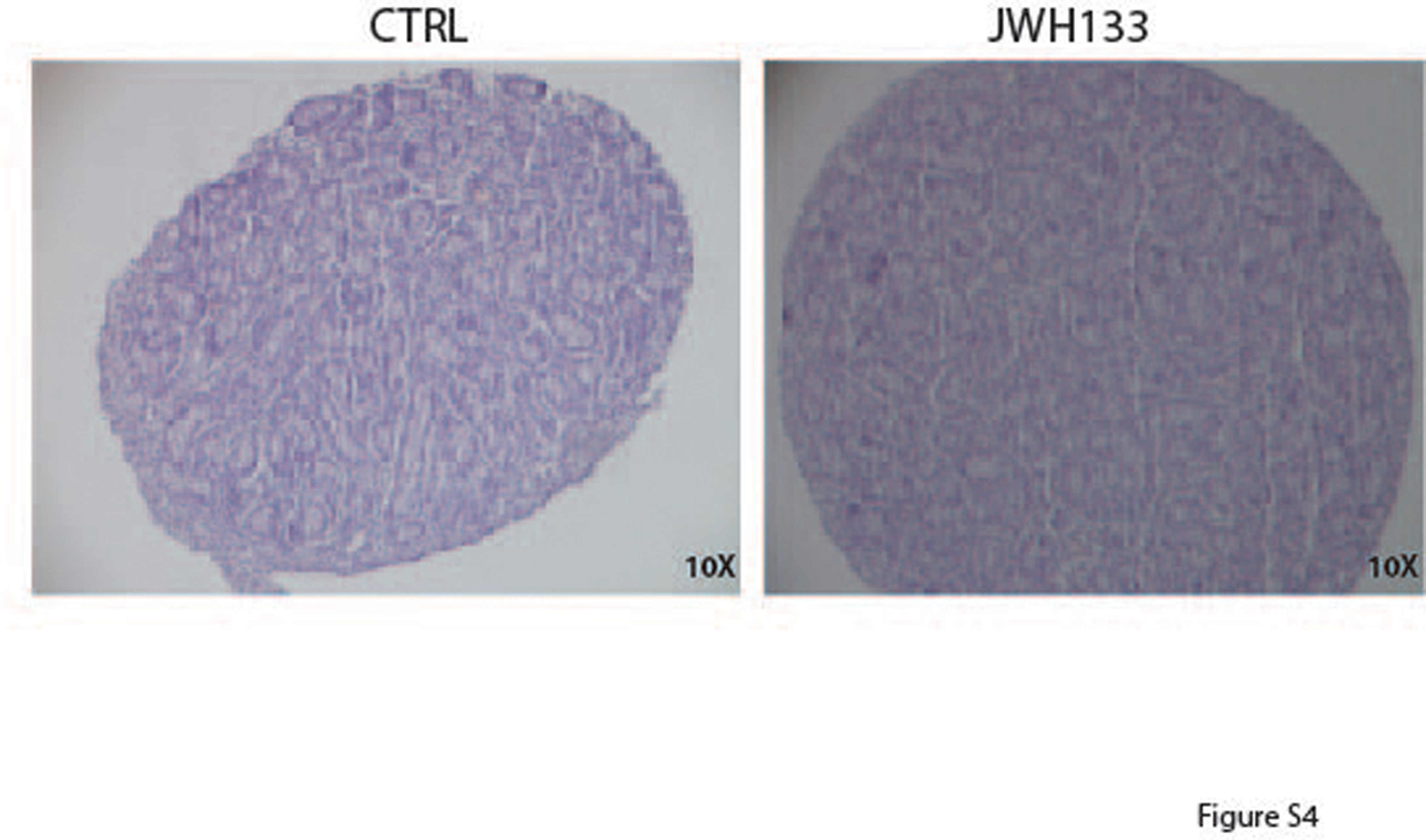

Supplement: Supplementary Figure S4 [file cddis2017496x5.tif]
